# An improved global database and model of plant available soil phosphorus

**DOI:** 10.1038/s41597-026-07140-3

**Published:** 2026-04-15

**Authors:** Richard W. McDowell, Alasdair D. L. Noble, Crile Doscher, Julian Helfenstein

**Affiliations:** 1 Faculty of Agriculture and Life Sciences, Lincoln University, Lincoln, New Zealand; 2 Bioeconomy Science Institute, Lincoln, New Zealand; 3 Soil Geography and Landscape Group, Wageningen University, 6700 AA Wageningen, The Netherlands

## Abstract

Plant-available phosphorus (P) is critical for plant production but can also be used an an indicator of P loss from soils and potential freshwater degradation. Global maps of plant-available P provide an overview of spatial patterns in plant-available P and are a critical input for various model applications, from agro-climate models to crop models. A previous map of Olsen P, the most widely applied soil test for plant-available P, was developed from ~575,000 observations, but informed by data biased toward Europe and North America. We present an updated and improved Olsen P dataset of 892,332 observations measured between 2000 and 2020. After filtering for consistent methods and depths we retained 42,034, greatly increasing geographic representation, particularly from Africa and Asia, and improving the balance of land uses sampled. Using these data, we applied a quantile random forest model with enhanced soil and land-use predictors to generate global grids (and maps) of the 20^th^, 50^th^ and 80^th^ percentile of Olsen P for the 0–20 cm depth at 1 km^2^ resolution. These data provide a more accurate, representative resource for assessing global patterns in fertiliser needs, modelling nutrient cycles, and evaluating potential freshwater P risks.

## Background & Summary

Plant-available soil phosphorus (P) is essential for global food production but also contributes to P losses that degrade freshwater quality^1^. Accurate maps of plant-available P are therefore critical for aligning fertiliser inputs with crop requirements^2^, preventing over-application, and reducing long-term soil P accumulation that elevates the risk of runoff into rivers and lakes^3^.

A previous global map of Olsen P, the most widely used indicator of plant-available P, was developed from filtered data at ~575,000 locations spanning cropland, intensively grazed grassland, rangeland, and forested areas^4^. This product has supported many assessments including of: the lifespan of global fertiliser reserves^5^; estimates of the large pool of unavailable P that may be accessed through crop breeding or management innovations^6^; and models of riverine P concentrations and algal bloom risk^7^.

Despite its utility, the original map can be strengthened by incorporating substantially more observations from under-represented regions (e.g., Africa and Asia), integrating additional soil-specific predictor variables, and providing clearer representations of uncertainty^8^. Here, we introduce an improved global Olsen P map that addresses these limitations by combining a more geographically balanced dataset with an enhanced modelling framework.

## Methods

Soil P data were compiled from a combination of global and regional databases, long-term trials, and international publications (Table 1). Some datasets were updated with new observations within their original period of interest (2000–2020)^4^, while others were newly available or recently published. Each source underwent a rigorous quality check for:

1. Soil extraction methods and plant-available P determination. Only data from widely used P extraction methods were included: Bray-I P, Colwell-P, Mehlich-3 P, Resin-P, and Olsen P. Data were restricted to gravimetric measurements above the detection limit of 1 mg kg^−1^. Volumetric data were excluded unless accompanied by bulk density information to allow conversion to gravimetric units.
2. Geographic coordinates and sampling date. Point data were verified for correct latitude and longitude, ensuring they were not located in waterbodies and free from common input errors (e.g., letters instead of numbers, missing negative signs for Southern Hemisphere locations). We prioritized samples measured between 2000–2020 and published by 2023. A small number of older observations (n = 146) were retained from long-term trials where land use and management were unchanged. Some of the new data (n = 1,068) were sourced from a study of P use efficiency in China^9^. This study contained 424 observations from 68 locations with publicly available coordinates and 813,890 points that were re-gridded, to maintain anonymity, from 169,978 observations measured in 2018. We took the mean of observations at each of 68 sites with known coordinates. Because the re-gridded data were provided at a finer spatial resolution (5 km²) than many of the predictors used in the global Olsen P model, we took a random sub-sample of 1,000 sites to maintain comparability with the number of sites with known locations in China (n = 68 + 783).
3. Data harmonisation. All non-Olsen P measurements were converted to Olsen P using established regressions (McDowell *et al*.^4^ for Bray-I P, Mehlich-3 P, and Resin P and Moody *et al*.^10^ for Colwell-P). Data were limited to samples whose upper depth was between 0−1 cm and lower depth was 10–20 cm, representing the plough layer in cropland or the main rooting zone in pastures^11,12^. Duplicate observations at the same coordinates were averaged.

**Table 1:** New sources of soil test P (methods other than Olsen are given in parentheses), geographic extent and number of samples before and after filtering/conversion.

Database	Geographic extent	Unfiltered observations	Filtered/converted observations	Refs.
Public databases				
World Soil Information Service Snapshot-2019	Global	7,231 (Mehlich-3)	748	^41^
		40,325 (Bray-I)	328	^41^
		12,287 (Olsen)	671	^41^
ISCRIC-WISE-2010	Global	2,189	1,147	^42^
Semi-natural soils	Global	2,865	1015	^43^
		1,157 (Bray-I)	384	^43^
Africa SoilGrids	Africa	49,225 (Mehlich-3)	2,111	^13^
Australian National Soil Information System	Australia	25,959 (Colwell)	500	^44^
		3713	93	^44^
Published studies with public soil test P data				
	Global	255	255	^45^
	Global	244	87	^46^
	Australia	15 (Colwell)	15	^47^
	Brazil	39 (Mehlich-3)	39	^48^
	China	169,978	1,000	^9^
		424	68	^9^
	Burkina Faso	67 (Resin)	67	^49^
	Ethiopia	66	66	^50^
	France	39	39	^51^
	Germany	53	53	^51^
	India	60	60	^52^
	Kazakhstan	7	7	^53^
	Thailand	65 (Bray-I)	65	^54^
	Rawanda	150	150	^55^
	Sao Tome	1 (Mehlich-3)	1	^55^
	Spain	33	33	^51^
	Sweden	34	34	^51^
	Switzerland	1 (Mehlich-3)	1	^51^
		57	57	^56^
Sum new data		316,538	9,093	
Sum of data from McDowell *et al*.^4^		575,794	32,941	^4^
Sum all data		892,332	42,034	

Links to the raw (open) data are either given in the referenced citation or as links to supplementary information in the published studies.

**Fig. 1:**
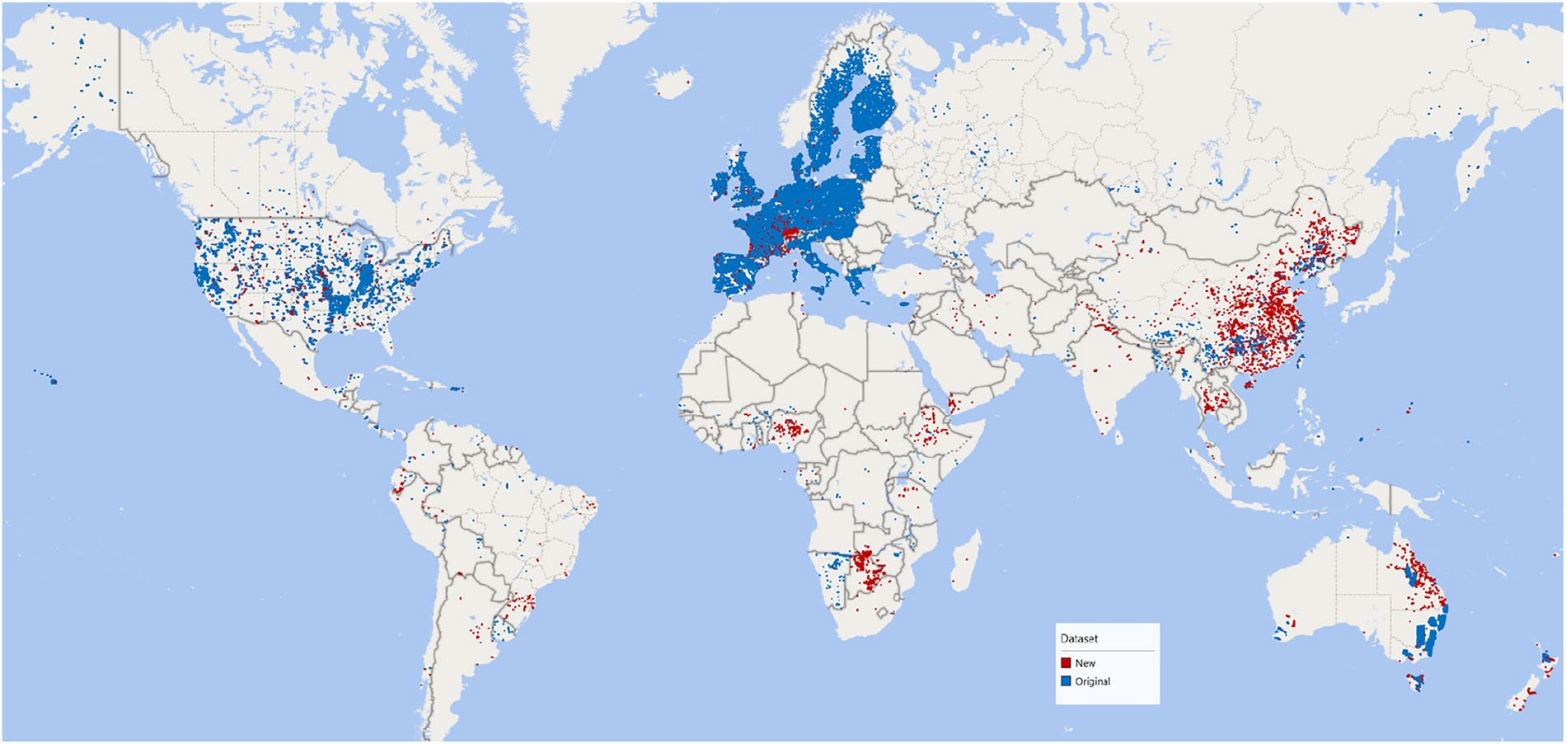
Location of samples with Olsen P data. The original data are in blue (n = 32,491) and new data are in red (n = 9,093). The base map used data sourced from OpenStreetMap contributors available under an Open Database License (https://www.openstreetmap.org/copyright).

The unfiltered dataset comprised 316,538 new points (Table 1). After filtering for method consistency, depth, and quality, 9,093 points remained (mean sampling depth 0–16 cm; Table 2), adding roughly a quarter more filtered observations to the original database (32,941 points). Notably, coverage increased by 650% in Africa and 687% in Asia, particularly in tropical regions (Table 2), with modest improvements in the Americas and Oceania. This substantially rebalances the dataset compared to the previous dominance of European and North American observations (Table 2; Fig. 1)^4^.

**Table 2:** Count, percentage increase, and median Olsen P concentration, and depth of sampling by continent.

Continent	Count	Percentage increase in count	Median Olsen P (mg kg^−1^)	Mean sampling depth (cm)
Africa	3,415 (525)	650	5 (5)	20 (19)
Asia	2,308 (336)	687	17 (8)	18 (18)
Europe	958 (16,684)	6	35 (30)	16 (20)
North America	1,443 (9,768)	15	10 (10)	12 (19)
Oceania	815 (5,482)	15	5 (5)	11 (19)
South America	154 (146)	105	7 (5)	14 (20)
Global	9,093 (32,941)	28	7 (17)	17 (20)

Values in parentheses refer to the original dataset published in McDowell *et al*.^4^.

### Modelling

The filtered Olsen P data were paired with predictor variables from diverse sources (Table 3), selected for their likely influence on soil P. These included climate, land use, soil chemical and physical properties, and ecological classifications^13,14^. To ensure all data were consistent, we extracted data for each predictor variable from the sources outlined in Table 3 at a resolution of 10 km^2^ for both the original dataset and new observations. This totalled 13,449,107 points, of which 7,167,304 points would be available if areas unlikely to be used for primary production (permafrost, desserts and mountains) were excluded^5^. Compared to the original analysis^4^, this study included more predictors describing soil parent material and physiochemical parameters, and fewer climate variables, reflecting the low importance of some climate predictors (e.g., monthly runoff) in previous models^15^.

**Table 3:** Climatic, biophysical and geographic variables and the units, years and sources of the variables used to predict the global Olsen P concentration.

Variable	Unit	Year measured	Source
Country	Name	2015	^57^
Mean annual temperature	°C	2015	^58^
Mean annual precipitation	mm	2015	^58^
Altitude	m above sea level	2010	^59^
Slope	%	2010	^59^
Soil bulk density (0–15 cm)	g cm^−3^	2016	^27^
Sand (0–15 cm)	%	2016	^27^
Silt (0–15 cm)	%	2016	^27^
Clay (0–15 cm)	%	2016	^27^
Soil pH (water, 0–15 cm)	—	2016	^27^
Soil organic carbon (0–15 cm)	g kg^−1^	2016	^27^
Soil nitrogen (0–15 cm)	%	2016	^27^
Non-anthropogenic total P (0–20 cm)	mg kg^−1^	2015	^60^
Soil volumetric water holding capacity (0–15 cm)	g cm^−3^ at −33 kpa	2016	^27^
Above ground grassland biomass	kg ha^−1^	2020	^61^
Soil cation exchange capacity (0–15 cm)	meq. 100 g^−1^	2016	^27^
Enhanced vegetation index (EVI, monthly)	EVI at a 1 km^2^ resolution	2010	^62^
Normalised difference vegetation index (NDVI, monthly)	NVDI at a 1 km^2^ resolution	2010	^62^
Mean annual runoff	mm	2014	^63^
Population density	Persons/km^2^	2015	^64^
Cropland	Class (yes/no)	2000	^65^
Percentage of forest	%	2015	^66^
Percentage of bare land	%	2015	^66^
Percentage of grassland or shrubland if not forestland, cropland or bare land	%	2015	^65–67^
World Reference Base soil group	Class	2016	^27^
Parent material	Class	2010	^68^
Income class	Class (low - high)		^57^
Catchment area	km^2^	2010	^69^
Biome	Class	2016	^70^

**Fig. 2:**
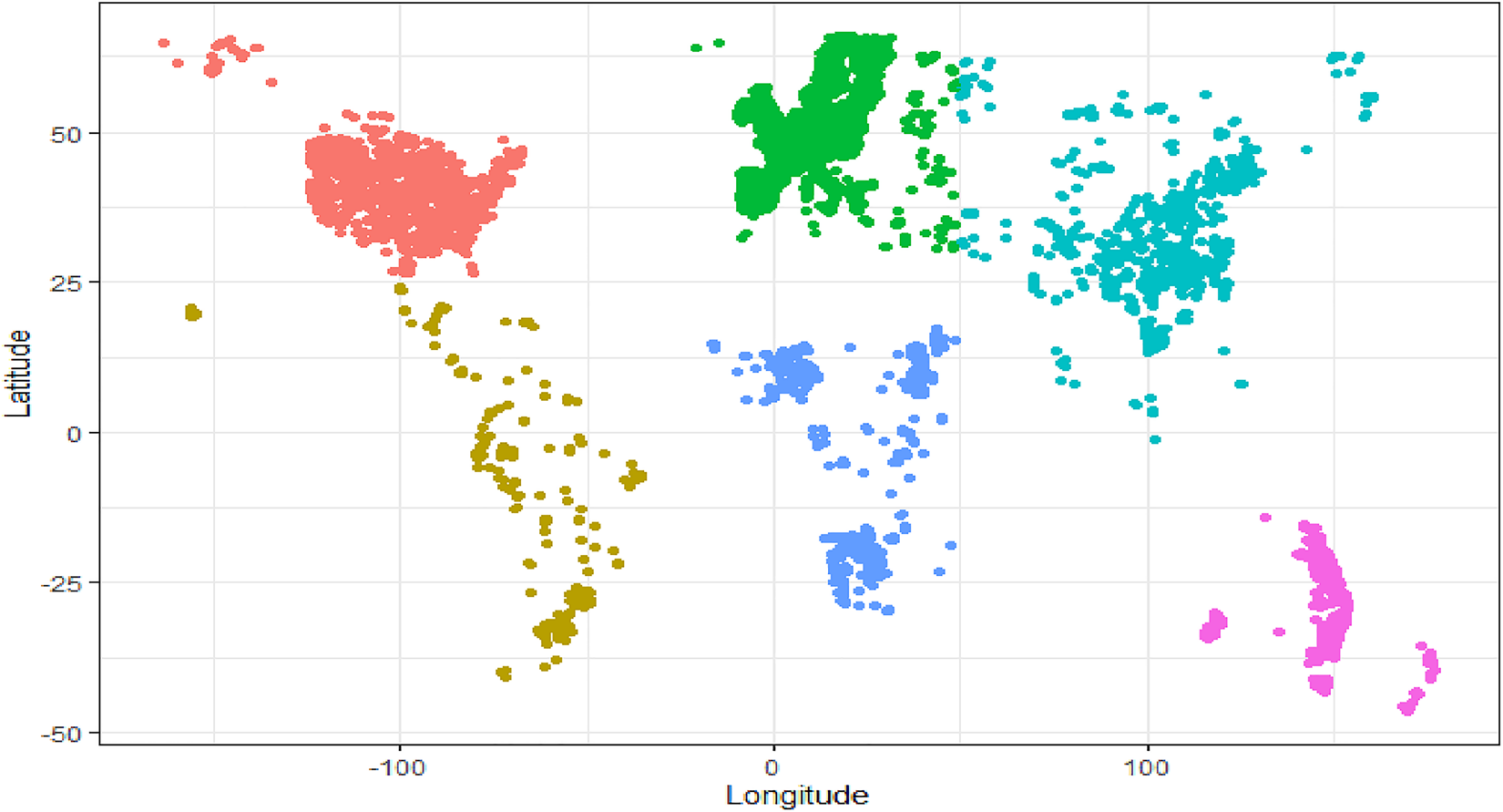
Plot of the geographic clustering of data by continent used when selecting data to train and test the models to minimise spatial autocorrelation.

We trialled a range of models to predict Olsen P concentration: classification and regression trees^16^, multivariate adaptive regression splines^17^, gradient boosted regression trees, training and test datasets were sampled to include equal proportions from each continent (Fig. 2).

**Fig. 3:**
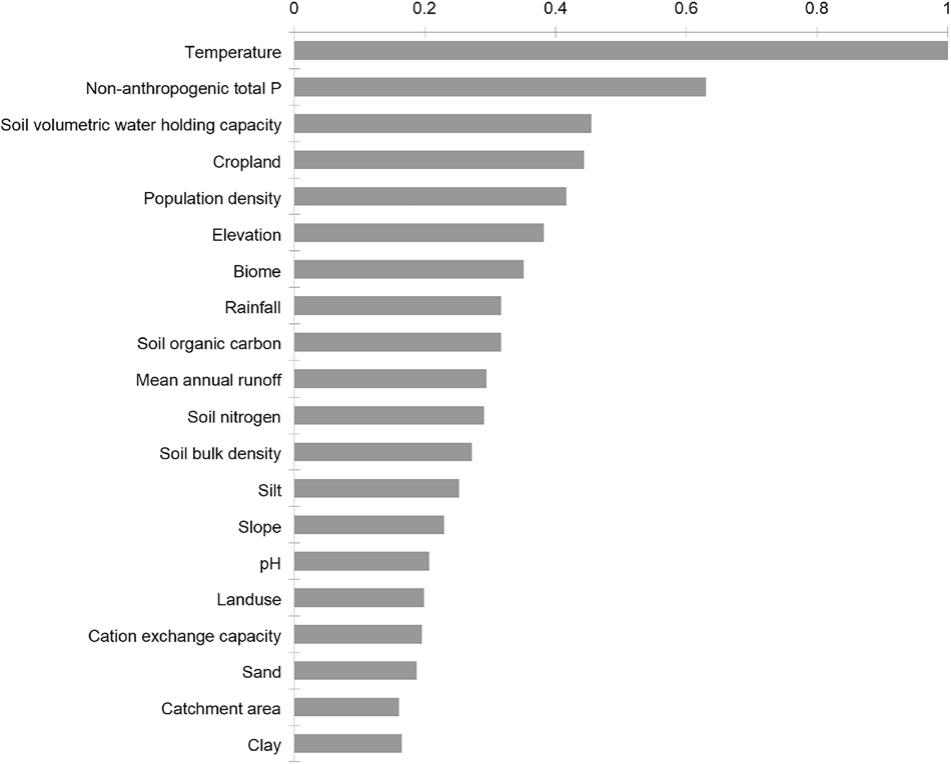
Relative importance of variables included in the quantile random forest model chosen to predict global Olsen P concentrations.

**Fig. 4:**
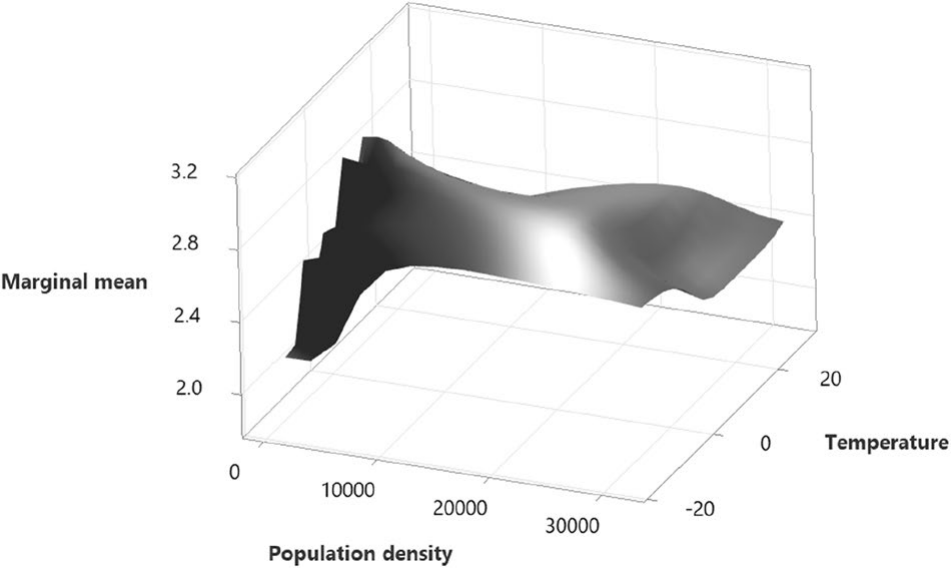
Marginal mean change in fit of natural log Olsen P concentrations with changes in population density and temperature.

Quantile random forest regression provided the best performance (Table 4) and was therefore selected for final mapping. As with all machine learning approaches, quantile random forests identify statistical associations rather than causal mechanisms among predictor variables^18^. Accordingly, the most important variables (Fig. 3) represented a coherent set of climatic (e.g., mean annual temperature and runoff), soil physical and fertility attributes (non-anthropogenic total P and soil nitrogen concentrations) and land use characteristics. Together these variables plausibly reflect the biophysical and socio-economic (e.g., population density and land use intensity) associated with the production of low to high P-requiring crops^5,19^. For example, higher temperatures and greater soil water-holding capacity can support expansion of cropland in regions with inherently fertile soils. These conditions are typically managed more intensively in areas of higher population density and shorter supply chains, where agricultural inputs and outputs are more tightly coupled. Importantly, although several predictors act synergistically, their interactions are not uniformly positive. In some cases, Olsen P increased only modestly, or not at all, due to interacting effects among variables. For instance, higher population density may initially be associated with increasing Olsen P concentrations, followed by stabilisation or decline at higher temperatures (Fig. 4), indicating non-linear and context-dependent associations, but not causal links^18^, captured by the model.

**Table 4:** Model performance for the prediction ln(Olsen P) concentration.

Model approach	Coefficient of determination	Root mean square error (Ln Olsen P)	Mean absolute error (Ln Olsen P)
Classification and regression trees,	0.52	0.771	0.571
Multivariate adaptive regression splines	0.41	0.855	0.644
Gradient boosted regression trees	0.55	0.753	0.564
Quantile random forest regression	0.68	0.633	0.462

After selecting quantile random forest regression as the best model, we retrained the model using the full dataset to leverage all available information for spatial prediction^20,21^. This approach improves predictive accuracy once the model’s structure, parameters and performance have been validated^22,23^. The 20^th^, 50^th^ and 80^th^ percentiles of predicted global Olsen P concentration were produced after correction for retransformation bias with the smearing estimate method:

1 $$S=\frac{1}{n}{\sum }_{i=1}^{n}{e}^{{\hat{s}}_{i}}$$

where *ε*_*i*_ denotes the residuals of the regression models. The correction factor (*S*) is applied over the whole range of predictions, as it is assumed that the residuals are homoscedastic.

The back-transformed predictions of Olsen P concentrations in topsoil P were projected globally in Q-GIS to a grid at a spatial resolution of 10-km^2^, which corresponds to the coarsest grid cells associated with the input data as listed in Table 3. Grids were then were interpolated using Nearest Neighbours and an Equal Earth projection (EPSG8857) at a spatial resolution of 30 arc-seconds (ca. 0.00833 degrees of 1 km^2^ near the equator), before presenting final geotiffs in a WGS84 (EPSG:4326) projection. Median Olsen P concentrations highlight regions enriched in P due to recent or historical fertiliser application, such as East Asia, Europe, and North America^24,25^). Gridded predictions of the 20, 50, and 80^th^ percentiles of concentrations at 10 km^2^ are available in Figshare^26^ for further analysis.

### Calculation of soil Olsen P stocks

**Fig. 5:**
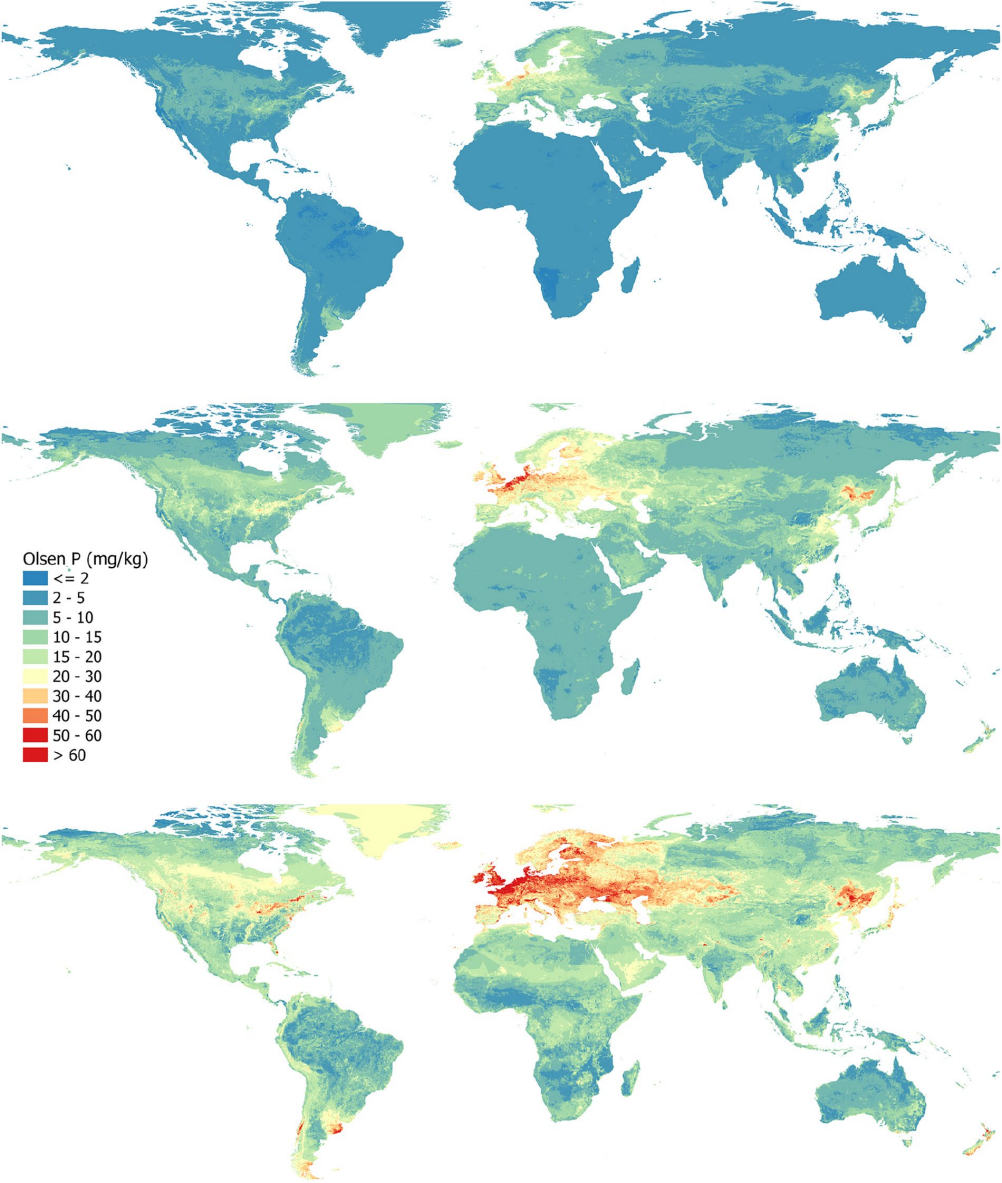
Example output of the 20^th^ (bottom) 50^th^ (middle) and 80^th^ (top) percentiles of global topsoil Olsen P concentration (mg kg^−1^). The mapped land parcels are interpolated using Nearest Neighbours to 1 km^2^ resolution from a grid at a 10 km^2^ spacing and predicted from a database containing *ca*. 722,000 soil samples of freely available data with a wide geographic coverage. The base map used data sourced from OpenStreetMap contributors available under an Open Database License (https://www.openstreetmap.org/copyright).

Global soil Olsen P stocks were calculated by multiplying predicted Olsen P concentrations (Fig. 5) by bulk density values^27^. Predictions and bulk density were assumed to represent 10 km^2^ land parcels with a topsoil depth of 20 cm. Stocks per pixel were expressed in kilotonnes (kt). The global Olsen P stock at the 50th percentile was estimated at 298,576 kt, with continental stocks ranging from 104,281 kt in Asia to 13,282 kt in Oceania (including Australia; Table 5). Mean Olsen P concentrations were highest in Europe (19.3 mg kg^−1^) and lowest in South America (6.2 mg kg^−1^). The global stock varied from 149,280 kt (20th percentile) to 676,751 kt (80th percentile; Table 5). Compared to the previous dataset, the improved model predicted that mean stock is approximately 6% lower, with the largest reduction observed in Europe.

**Table 5:** Median concentrations and total stock of Olsen P (50^th^ percentile; and 20^th^ and 80^th^ percentiles in parentheses) by continent, compared to previous estimates^4^.

Continent	Concentration (mg kg^−1^)	Stock (kt)	Previous estimates made in 2023 (kt)
Africa	6.7 (3.1–15.4)	54,954 (25,993–133,910)	47,847
Asia	8 (3.8–18.7)	104,281 (49,324–239,820)	86,474
Europe	19.3 (9.8–41.6)	46,389 (25,175–92,819)	84,401
North America	8.8 (4.3–21.3)	50,762 (26,337–111,612)	60,517
Oceania	7.8 (3.3–18.9)	13,282 (7,672–28,745)	13,374
South America	6.2 (3.1–15.4)	28,908 (14,779–69,845)	26,005
World	7.9 (3.8–18.4)	298,576 (149,280–676,751)	318,618

## Data Records

All data are deposited as CSVs containing the Olsen P data (“V2_Olsen_P_original_data.csv”) and global predictors in a 10 km^2^ grid (“V2_Global10k2_OlsenP_Predictions.zip”), an Excel spreadsheet of the filtered and unfiltered data (Table 1; “V2 Final Olsen P.xlsx”), and GeoTIFF maps of the median and 20^th^ and 80^th^ percentiles of predicted Olsen P concentration (“V2_20^th^.zip”, “V2_50^th^.zip”, and “V2_80^th^.zip”) calculated using the Random Forest quantile regression model (“V2_OP_For_Paper.r”) are in the Figshare repository^26^. The original data citation has been updated to direct users to this enhanced dataset.

## Technical Validation

### Validating conversions to Olsen P

**Fig. 6:**
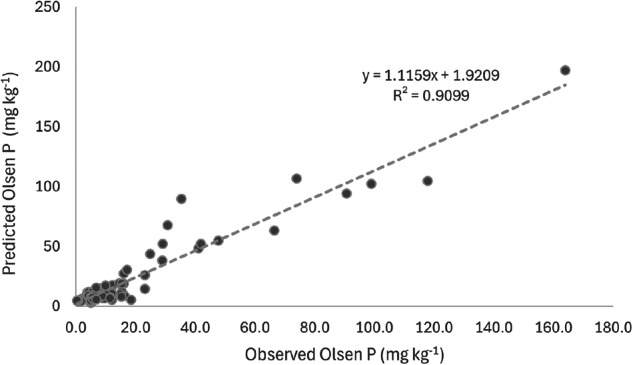
Validation of the conversion of Colwell P into Olsen P using the conversion equation of Moody *et al*.^10^ and independently collected Olsen P data measured from sites across Australia.

Previous conversions from Mehlich-3 P and Bray-I P to Olsen P were validated using independent data from the National Cooperative Soil Survey (97 samples)^4^. We validated the Colwell P to Olsen P conversion using Moody *et al*.^10^ with 90 soils from independent Australian studies^28–31^. Predictions for Olsen P using the Colwell data and conversion equation of Moody *et al*.^10^ yielded predictions whose liner regression had a slope and intercept (Fig. 6) not significantly different from 1 and zero, respectively indicating converted data to be robust. Nevertheless, some uncertainty remains due to inter-laboratory variability in analytical procedures, even within the same extraction method.

### Olsen P stock validation

We compared our estimate of the sum of the 50^th^ percentile of Olsen P stock in sub-Saharan Africa to the previously modelled and published P stock in Sub-Saharan Africa. Excluding the Saharan Desert, our modelled estimate was 26,888 kt of Olsen P for the 0–20 cm depth (ranging from 14,147 to 64,671 kt for the 20^th^ and 80^th^ percentile). When converted into Olsen P from Mehlich-3 P (estimated for the 0–30 cm depth), the previously published stock of 28,890 kt^32^ fell close to our estimate of the Olsen P stock for Sub-Saharan Africa.

### Validation of geographic performance

**Fig. 7:**
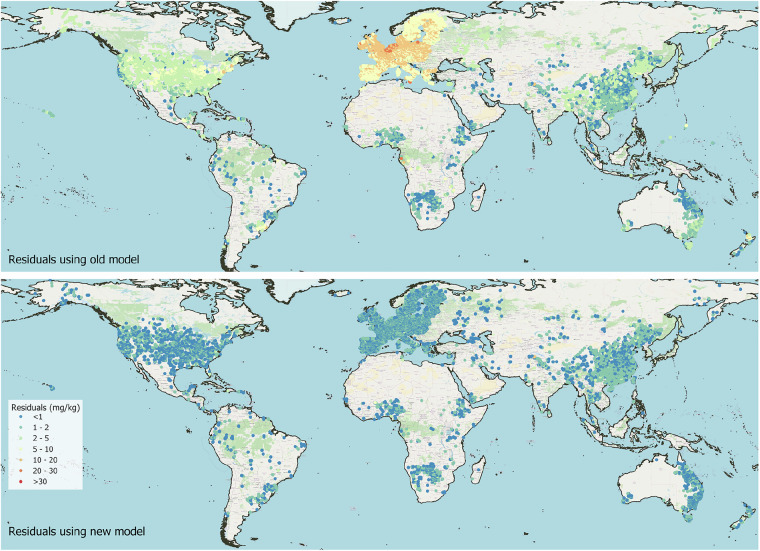
Map of the residuals for each data point calculated as the difference between observed and predicted concentrations (mg kg^−1^) using the old (generalised additive) and new (quantile random forest) models. The base map used data sourced from OpenStreetMap contributors available under an Open Database License (https://www.openstreetmap.org/copyright)^70^.

Residuals (predicted – observed) were reduced from 6.7% in the old model^4^ to 1% in the new quantile random forest model. Absolute residuals were classified (<1, 1–2, 2–5, 5–10, 10–20, 20–30, >30 mg kg^−1^). The percentage of residuals in the old model in each class was 2, 11, 25, 24, 20, 1 and <1%, respectively. For the new model, the percentage in each class was 53, 46, 1, <1, <1, <1, and <1%, respectively. Maps showing the geographic spread of the residuals for the old and new models are in Fig. 7. The new model had fewer large residuals (Fig. 7), improving predictions in regions previously over-represented (e.g., Europe and North America). Consequently, predicted Olsen P stocks in Europe were about 40% lower than previous estimates (Table 5).

## Usage Notes

These concentration data can be used to characterise broad, global patterns in soil Olsen P, for example as inputs to global vegetation, biogeochemical, or crop models^33–35^. They can also be used to assess whether Olsen P concentrations are above or below crop-specific threshold values required for optimal growth now and under a changing climate^36,37^. Such assessments can inform strategic decisions on phosphorus (P) fertiliser use, whether to apply P to reach or maintain threshold concentrations or to reduce or cease applications, thereby lowering costs, supporting circular nutrient use and manure recycling, and reducing the risk of P losses to surface waters and eutrophication^1,38–40^. More efficient use of P also helps extend finite fertiliser stocks and global P reserves^6^.

Despite improved spatial coverage and predictive performance, we recommend that these data and model outputs be applied at relatively coarse spatial scales (e.g. catchments), as uncertainty in point-level predictions remains high due to strong soil heterogeneity and data gaps in some regions. Furthermore, because the underlying data span multiple years and the predictions do not explicitly account for fertiliser or manure inputs, site-specific fertiliser recommendations and long-term soil P stock assessments should be supported by contemporary, local soil sampling.

## Data Availability

The data are available in Figshare (https://figshare.com/s/185ef41e084140738e0b)^26^. Please note that this link is to an updated repository with version 2 of the database, code and GeoTIFFs. No bespoke code was produced during the analysis. However, all the data generated during each processing step, including the quantile Random Forest model (“V2_OP_For_Paper.r”), are available in the updated Figshare repository^26^.
